# Photoplethysmography-Based Distance Estimation for True Wireless Stereo

**DOI:** 10.3390/mi14020252

**Published:** 2023-01-19

**Authors:** Youngwoo Jeong, Joungmin Park, Sun Beom Kwon, Seung Eun Lee

**Affiliations:** Department of Electronic Engineering, Seoul National University of Science and Technology, Seoul 01811, Republic of Korea

**Keywords:** true wireless stereo (TWS), photoplethysmography (PPG), distance estimation, waveform adjustment (WA), noise reduction, embedded system

## Abstract

Recently, supplying healthcare services with wearable devices has been investigated. To realize this for true wireless stereo (TWS), which has limited resources (e.g. space, power consumption, and area), implementing multiple functions with one sensor simultaneously is required. The Photoplethysmography (PPG) sensor is a representative healthcare sensor that measures repeated data according to the heart rate. However, since the PPG data are biological, they are influenced by motion artifact and subject characteristics. Hence, noise reduction is needed for PPG data. In this paper, we propose the distance estimation algorithm for PPG signals of TWS. For distance estimation, we designed a waveform adjustment (WA) filter that minimizes noise while maintaining the relationship between before and after data, a lightweight deep learning model called MobileNet, and a PPG monitoring testbed. The number of criteria for distance estimation was set to three. In order to verify the proposed algorithm, we compared several metrics with other filters and AI models. The highest accuracy, precision, recall, and f1 score of the proposed algorithm were 92.5%, 92.6%, 92.8%, and 0.927, respectively, when the signal length was 15. Experimental results of other algorithms showed higher metrics than the proposed algorithm in some cases, but the proposed model showed the fastest inference time.

## 1. Introduction

Wearable devices have garnered an increasingly significant attention owing to their various applications [1,2]. Among them, smart watches provide healthcare services, such as measuring body composition and heart rate, by employing electrocardiography (ECG), bioelectrical impedance analysis (BIA), and photoplethysmography (PPG) sensors [3,4]. In contrast, true wireless stereo (TWS) does not provide healthcare service yet although heart rate and oxygen saturation is feasible to measure at the arterial blood of the outer ear. To provide the healthcare services with TWS, there is a need to mount a new biosensor but it is difficult as TWS allows only small areas. Therefore, there is a need to provide multiple functions simultaneously with one sensor.

PPG sensors are widely employed in wearable devices [5,6]. PPG sensors emit infrared rays to the skin and measure the amount of blood flow by determining the amount of rays absorbed in red blood cells. Because the PPG data are affected by heart rate due to this operation method, PPG sensors provide healthcare services such as heart rate measurement, breathing rate estimation, atrial fibrillation, and blood pressure measurement [7,8,9,10]. In addition, since heart rate has specific patterns, applying the deep learning for pattern recognition on PPG signal was researched [11,12]. The atrial fibrillation detection with the hybrid model of convolutional neural network (CNN) and recurrent neural network (RNN) achieved accuracy of over 99% [13]. The DeepCNAP model for heart rate measurement using PPG signals was presented [14]. A deep learning model for robust PPG wave detection was proposed in [5]. The best performing model was a CNN-long short-term memory (LSTM) algorithm with a PPG synchro-squeezed Fourier transform (SSFT) and the accuracy, precision, and recall were 0.894, 0.923, and 0.914, respectively.

The PPG data are also influenced by the subject’s skin characteristic and motion artifact; these factors make raw data produce noise [6,15,16]. In order to reduce the noise of signal, a motion reduction technique for respiratory rate was proposed [17]. The proposed technique reduced motion interference by removing similar spectra with an accelerometer sensor and an adaptive filter. By applying the technique to the raw PPG data, a clear spectrum was produced. Similarly, extracting heart rate and respiration rate values from raw PPG data with a three-axis accelerometer for motion reference was studied [18]. This study proposed an adaptive notch-filtration architecture, which comprises the adaptive moving average filter, the adaptive notch filter, and the extraction for physiological parameters. With the proposed filter, the filtered PPG signals for the calculation of the heart rate and respiratory rate were similar to measurements from commercial devices for the IEEE-SPC dataset and the in-house dataset. For the noise reduction, the enhanced empirical wavelet transform algorithm was proposed [19]. This algorithm employs a fast Fourier transform and the order statistical filter. Compared with other conventional methods, the proposed method shows the best accuracy.

On the one hand, in addition to studies that employ the raw PPG data [7], studies that reduce the instability of the raw PPG data by applying several filters have been conducted [20,21,22,23,24,25,26]. Multi-mode particle filtering methods that demonstrate the performance improvement of an average error of less than 2 BPM compared to single-mode particle filtering and advanced methods with approximately 47 PPG recordings were introduced [23]. Two cutting-edge pulse detection algorithms on actual raw PPG data were studied [24]. This work demonstrated the effect of preprocessing pulse peak positions and the performance of peak detection algorithm was analyzed on 21,806 pulse data [25]. Meanwhile, a study on the data compression method with stochastic modeling for power efficiency was performed [26]. The method that models the single cardiac period of PPG waveform applying two sets of Gaussian functions on the forward and backward wave of PPG pulse outperformed conventional delta-modulation-based methods.

**Figure 1:**
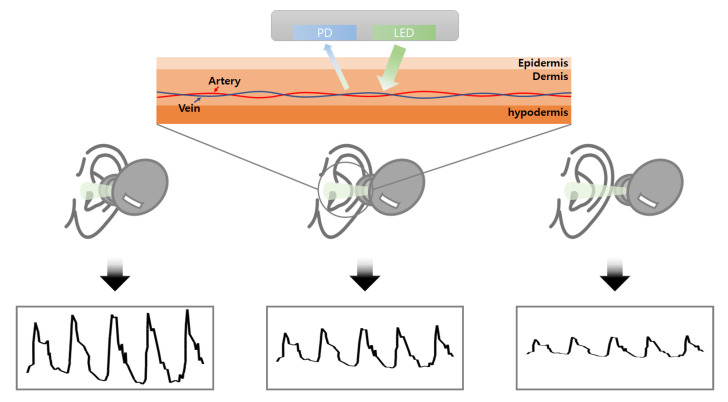
Mechanism of PPG sensor according to the distances between user and the sensor.

In this paper, we propose a distance estimation algorithm between the user and the sensor based on a waveform adjustment (WA) filter for the PPG data of TWS. By mounting the PPG sensor on TWS, various healthcare services were implemented with one sensor. However, because of the size limitation of the TWS, the existing sensor has to be removed when the PPG sensor is built into the TWS. Accordingly, the PPG sensor is responsible for the function that was implemented with the removed sensor. Figure 1 shows the working principle of the PPG sensor mounted on TWS. As the PPG data output the amplitude value, the data are different according to the distances between user and sensor. The existing distance estimation function of the wearable device is replaced by utilizing this characteristic of the PPG data. We designed a PPG monitoring testbed for collecting analog PPG signals and a signal processing logic for distance estimation. Owing to the instability of the PPG signal, the distance estimation logic includes the filter for noise reduction. We developed our PPG dataset according to the three criteria for distance estimation. The dataset was trained on various machine learning models, and we analyzed the performance of each model according to the inference result. The highest accuracy was 92.5% with the proposed model when the signal length was 15.

The contributions of this paper are as follows. To the best of our knowledge, it is the first work that proposes distance estimation with a PPG sensor. In order to provide healthcare services by mounting PPG sensors on the TWS, we designed a distance estimation algorithm to increase the area efficiency of the TWS by replacing a sensor for existing distance estimation to the PPG sensor. Digital filter IP and an analog-to-digital converter (ADC) controller were designed with Verilog HDL and implemented on field-programmable gate array (FPGA).

The remainder of this paper is organized as follows. In Section 2, we introduce the system architecture of the distance estimation for the PPG sensor, which includes the function for waveform adjustment and MobileNet, which is a lightweight deep-learning model. Section 3 presents the flow of the proposed algorithm. Section 4 explains the implementation of the proposed algorithm and analyzes the results. Finally, a discussion is provided in Section 5.

## 2. System Architecture

**Figure 2:**
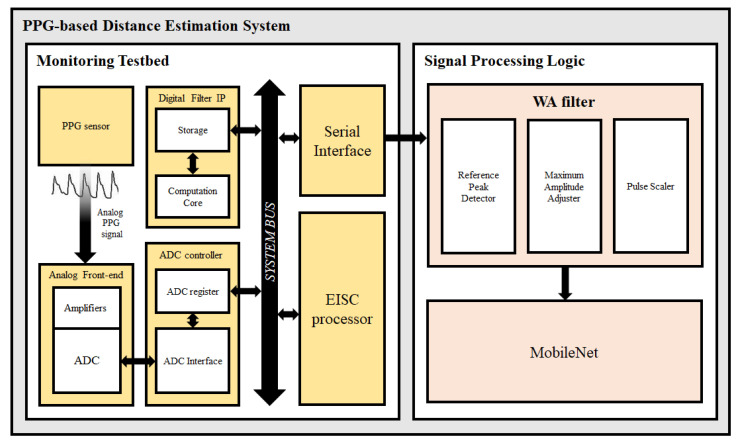
Block diagram of the proposed system.

Figure 2 presents the block diagram of the distance estimation system for the PPG sensor. The proposed system comprises the PPG signal monitoring testbed with hardware implementation and estimation logic is realized using a software.

### 2.1. PPG Monitoring Testbed

The PPG monitoring testbed includes an analog front-end for receiving the analog PPG signal, ADC controller, digital filter IP, extendable instruction set computer (EISC) processor, and a serial interface for data transmission [27]. The analog front-end contains an amplifier for the PPG sensor and an ADC for converting the analog signals to digital data. The storage module stores the parameters, input signals, output signals, and delayed samples. The EISC processor is the main core of the testbed, which is a 32-bit embedded processor with a three-stage pipeline and Harvard architecture. Because the low-power operation and area efficiency are important for the TWS, a floating point unit is not included in the processor. Accordingly, the testbed requires a signal processing unit for the analog PPG signal. There are two digital filter types: finite impulse response (FIR) and infinite impulse response (IIR) filters. The characteristic of the FIR filter is the absence of a regression component. Hence, a large amount of resources is significantly required as the order of the FIR filter increases. However, the IIR filter does not require substantial resources because the formula in which the values of the input and output signals are recursively applied is repetitive. For the power efficiency of the proposed system, we adopted the IIR filter. The parameter, input, output, and delayed samples of the IIR filter are stored in the storage module. The main processor converts the analog PPG signal to the digital PPG signal by controlling the registers of the ADC controller and transmitting them to the estimation logic.

### 2.2. Signal Processing Logic

Deep learning extracts appropriate features by stacking layers and updating the weight of the kernel (filter). In particular, because the process of extracting features implicates the spatial information on data with windows corresponding to the kernel sizes, it is important to maintain the spatial information of data by implicating the relationship between the elements of input data [28].

One-dimensional PPG signal data, which exist sequentially based on the time axis, have feature information related to pulse patterns from spatial information of the front and rear data according to signals that change by blood flow. However, when measuring the distance from the body surface with the PPG sensor, the amplitude sections of the PPG signals differ because the physical structure is different for each experimental group. In addition, various factors, such as light refraction or scattering due to the space created between the PPG sensor and the body surface in a specific distance section, distort the raw signal. Owing to such signal distortion, it is difficult to comprehensively extract only features related to the pulse pattern of a PPG signal, which is a bio-signal. Therefore, by keeping a pulse pattern that implies spatial information and scaling to the estimated amplitude range according to the specific distance section, the difference in the amplitude intervals triggered by the characteristics of the bio-structure is discarded. The signal processing logic, which estimates the distance, comprises a WA filter for waveform correction and MobileNet for training and inference.

#### 2.2.1. Influence Differential Distribution (IDD) Function

In the inference process, there is no information on the estimated amplitude range. Therefore, we determine the maximum amplitude with 99% confidence interval in the signals collected at a certain time and adjust the estimated maximum amplitude within the range of the estimated amplitude over a certain distance section using the function we designed. The IDD function adjusts the position of a sample point via the influence of reference points in a one-dimensional space. This influence depends on the distance between each reference point and sample point, and becomes stronger as the distance decreases, similar to the gravity between objects. The function repositions the sample points in the appropriate range by exerting influence, which depends on the distance from a sample point.

**Figure 3:**
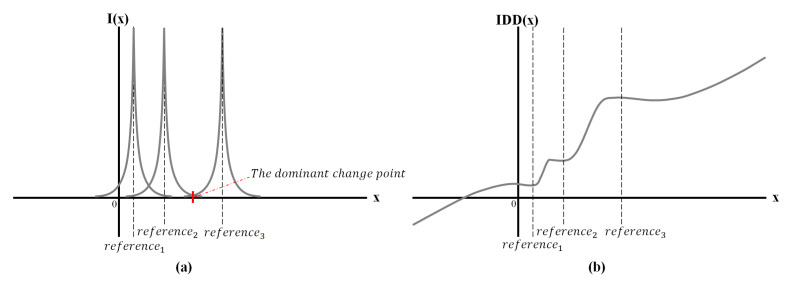
(**a**) Influence of the three reference points (**b**) Adjustment of sample points according to IDD function.

The IDD function is expressed by the influence equation *I*(*x*) for the variable *x* representing the sample point. Equation (1) represents the influence equation and the constant *r* represents the reference point of influence. The equation is expressed as having a positive value by squaring the distance between reference point *r* and sample point *x*; log is taken to scale the value since the range of variation of the actual input amplitude value is large. In addition, the result of influence equation decreases rapidly as the difference in the distances increases by using the exponential function. Equation (2) expresses Equation (1) as the inverse of the distance. According to the equation, if variable *x* is the same as constant *r*, the result of the influence goes to infinity. This feature allows all other influences to be ignored at the reference point. Figure 3a presents a graph illustrating each influence function of the three reference points.

(1) $$I\left(x\right)=e^{-\log_{2}{\left(r-x\right)}^{2}}$$

(2) $$I\left(x\right)={\left(\frac{1}{{\left(r-x\right)}^{2}}\right)}^{\frac{1}{\ln2}}$$

Equation (3) elucidates the influence coefficient (*IC*) normalized to a value between 0 and 1 for the influence of each reference at a specific point when multiple references exist. Equation (4) defines the maximum *IC* at that point. In addition, the maximum influence changes at the midpoint of the two close reference points, which is called “the dominant change point” in Figure 3a.

(3) $$IC_{i}=\frac{I_{i}}{\sum (I_{i})}$$

(4) $$IC_{max}=\max\left(IC_{i}\right)$$

The estimated adjustment point (*EAP*) is expressed by multiplying each reference point by the *IC* ratio and adding all of them, as shown in Equation (5). However, this adjustment equation has a problem in that the sample point is dragged to the reference point rapidly when the sample point is closer to the maximum influence. Therefore, we calculated the improved adjustment point using the maximum *IC*.

(5) $$EAP=\sum (IC_{i}\times r_{i})$$

To solve the problem of strong attraction to the reference point, the improved adjustment point is computed using Equation (6), which adds the *EAP* and sample point *x* as a specific ratio of the maximum *IC*. We named this equation the IDD function. Figure 3b, an example of the three reference points, verifies that the slope around each reference point decreases significantly and a value close to the reference point emerges around the reference point. Hence, the position of a sample point is readjusted appropriately around the reference point by the influence of each differentially distributed reference point.

(6) $$IDD\left(x\right)=IC_{max}\times EAP+\left(1-IC_{max}\right)\times x$$

#### 2.2.2. WA Filter

**Figure 4:**
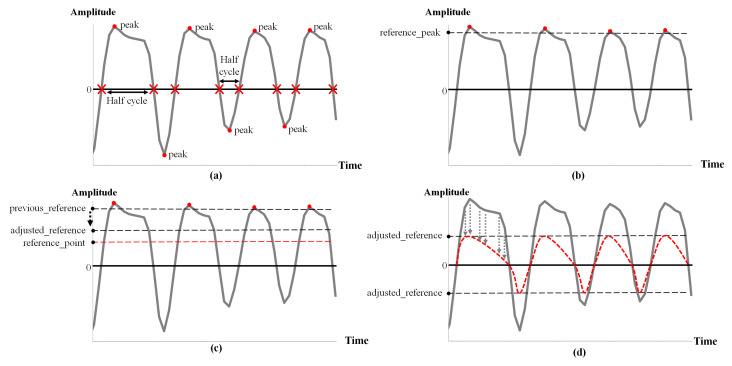
(**a**) Half-cycle detections of the PPG signal; (**b**) Distribution of the reference peaks; (**c**) Adjusted reference point; (**d**) Results of the scaled PPG signal.

The WA filter comprises a reference peak detector, maximum amplitude adjuster, and pulse scaler. The reference peak detector extracts the sign change points on the PPG signals of a certain period (=signal length) by ascertaining whether multiplying the results of consecutive sampling points is less than zero. This block regards a gap between a sign change point and the sign change point that follows it as a half cycle, as shown in Figure 4a. In addition, the maximum value of the half cycle is determined as a peak. In the next step, the reference peak is set as the 99% confidence interval for each peak distribution multiplied by the root of the total number of peaks, as illustrated in Figure 4b. The value *n* in Equation (7) represents the total number of peaks.

(7) $$reference\_peak=\left(2.58\times \frac{\sigma }{\sqrt{n}}\right)\times \left(\sqrt{n}\right)$$

The maximum amplitude adjuster fine tunes the reference peak using the IDD function. Because the reference peaks differ depending on the distance between the user and the sensor, we set the expected reference peak corresponding to the 95% confidence interval in the training data for three criteria for distance estimation. Finally, the reference peak is adjusted by applying the expected reference point for each distance to the IDD function, which is described in Figure 4c. Pulse scaler is a block that filters the PPG signal last before training or inferencing the AI model. Figure 4d demonstrates that each pulse is scaled at the ratio of the previous peak to the adjusted peak.

#### 2.2.3. MobileNet

**Figure 5:**
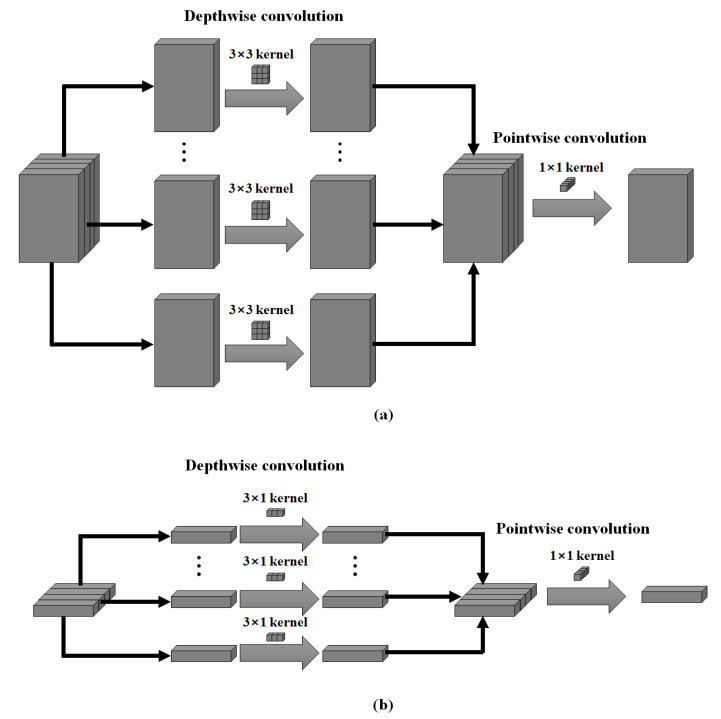
(**a**) Original architecture of MobileNet; (**b**) Modified MobileNet architecture for the PPG signal.

MobileNet is a lightweight-focused model that employs depthwise separable convolution to apply deep learning in low-capacity memory environments, such as mobile phones and embedded systems [29]. Depthwise separable convolution is a form in which pointwise convolution is combined after depthwise convolution, as illustrated in Figure 5a. Depthwise convolution has an independent 3 × 3 kernel for each input channel, thereby creating a feature map equal to the number of input channels; the pointwise convolution then calculates the cross-channel correlation by applying 1 × 1 convolution to all feature maps created in depthwise convolution. Unlike a network with a general convolution architecture, this architecture reduces the number of parameters. Based on these features, we increased the channel by conducting depthwise separable convolution on a single dimension to enable training and inference on a single-channel PPG signal, and altered the size of the kernel used for depthwise convolution to 3 × 1, as shown in Figure 5b.

## 3. Algorithm Flow

**Figure 6:**
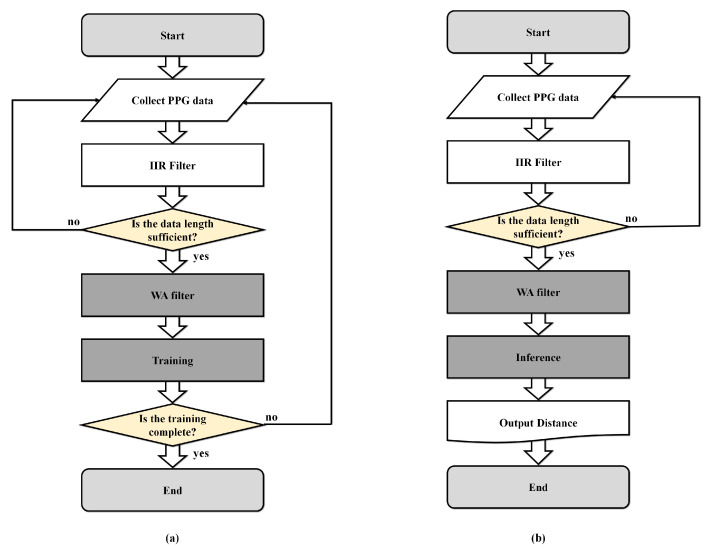
(**a**) Flow chart for training; (**b**) Flow chart for inference.

Figure 6 presents the proposed algorithm, which is divided into training and inference for MobileNet. The training procedure is presented as follows. First, the raw PPG signal is measured in real-time and the sample points are stored after IIR filtering. For the WA filter, as certain cycles of the PPG data are required, the previous process is repeated until a sufficient length of data is accumulated. When the primary data collection is completed, the waveform is normalized using a WA filter. In addition, because the minimum length of the filtered data is required for MobileNet, the process is performed several times. After securing sufficient data, MobileNet computes the optimum weight value, which is called training. The inference process is the same up to the data collection for MobileNet. Subsequently, the distances between the user and the sensor are estimated for the filtered data. We set the three criteria for distance estimation to 0 mm, 0.4 mm, and 0.8 mm, and MobileNet outputs one of these distances as the result.

## 4. Experiment

**Figure 7:**
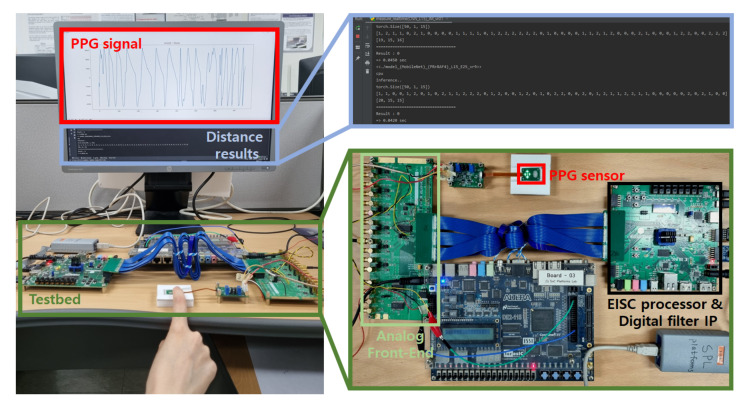
Experimental environment.

Figure 7 illustrates the experimental environment. Xilinx’s FPGA development board called Artix-7 was utilized for EISC processor and digital filter IP. We collected the PPG dataset with the monitoring testbed. The total dataset comprised 144,000 sampling points, and we collected 600 s twice from one person, and another 600 s from six people for each criterion of the distance estimation. To reduce the similarity between the datasets, 1500 sampling points from 500 to 1999 out of 6000 sampling points were set as a training dataset, and 3500 sampling points from 2000 to 5499 were set as an inference dataset. Accordingly, the total number of sampling points of the training and inference datasets are 36,000 and 84,000, respectively.

In order to verify the WA filter for the PPG signal, we designed the Kalman filter, short-time Fourier transform (STFT), modified average filter, bandpass filter (BPF)+single moving average (SMA) filter for performance analysis. The Kalman filter is a recursive algorithm that estimates unknown variables with previous and present data via noise reduction [30]. When the motion and measurement models are linear in the Gaussian distribution, this filter is available. The Kalman filter process comprises prediction and update steps. In the prediction step, the prediction vector is calculated using the motion model and the previous state vector. In the update step, the Kalman gain is updated by the difference of the prediction and measurement vectors, and is utilized to determine the state vector. Using this recursive process, the Kalman filter represents the state vector as the denoised data.

STFT is a filter for the audio signal process [31]. We expect the STFT to be appropriate for the PPG signal because the distribution of frequency for time was computed. STFT performs the Fourier transform while moving a window with a specific length in the signal. In this case, the Fourier transform is calculated several times for a specific time, and the frequency spectrum at that specific time is obtained by averaging the calculated timed. The most influential variable is the window length. It is important to set the proper window size because the resolution of the frequency domain decreases if the window length is short, and the resolution of the time domain decreases if the window length is long. Hence, we determined the window length to number 60 of the sampling points.

The modified average filter is a filter for noise reduction. Because amplitude fluctuation, due to signal bouncing, is fatal in estimating the distance between the user and sensor, we tried to correct bounced PPG signals based on the average. Accordingly, among the sampling points of the PPG signal, values of 2 times more and less than 1/2 of the average are regarded as incorrect data and replaced with averages. However, as this filter discards the relationship between the data before and after, the performance analysis on the accuracy demonstrates the importance of the relationship in the proposed algorithm. The modified average filter is employed in verifying that the distance estimation is feasible only with noise reduction.

The BPF+SMA filter is a hybrid filter for noise reduction. BPF discards noise by passing only a specific frequency band. The SMA filter utilizes a mean of previous data. Because the number of previous data increases, SMA becomes less sensitive to changes in the data and more robust to noise. In contrast, SMA becomes more sensitive to changes and less robust to noise when the number of previous data decreases. Therefore, the BPF+SMA filter is more effective in denoising than the single filter.

**Figure 8:**
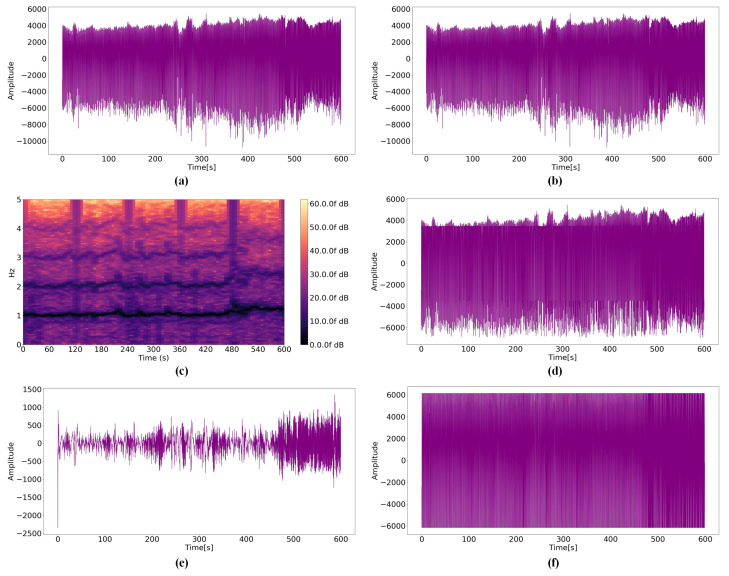
PPG signals for a distance 0 mm: (**a**) Raw data; (**b**) Kalman filter; (**c**) STFT; (**d**) Modified average filter; (**e**) BPS+SMA filter; (**f**) WA filter.

**Figure 9:**
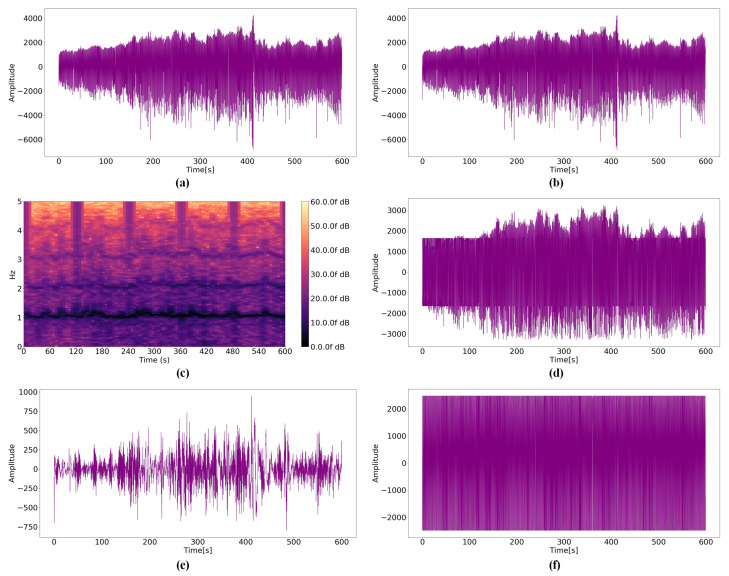
PPG signals for a distance 0.4 mm: (**a**) Raw data; (**b**) Kalman filter; (**c**) STFT; (**d**) Modified average filter; (**e**) BPS+SMA filter; (**f**) WA filter.

**Figure 10:**
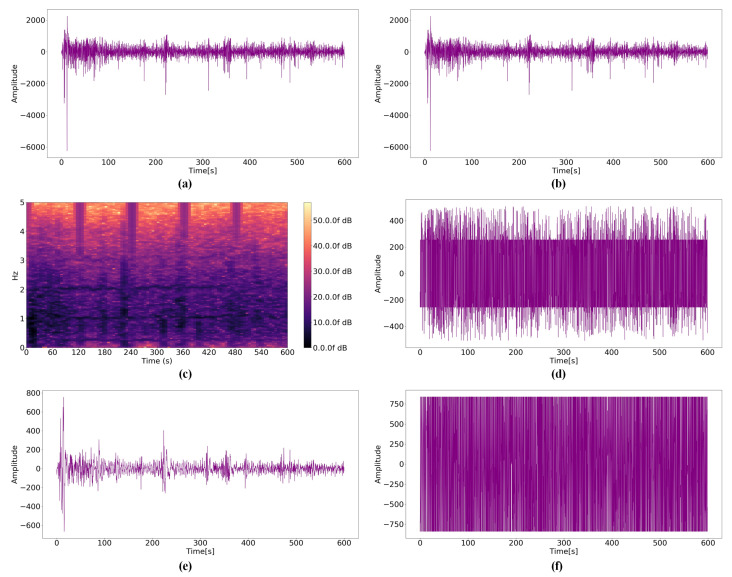
PPG signals for a distance 0.8 mm: (**a**) Raw data; (**b**) Kalman filter; (**c**) STFT; (**d**) Modified average filter; (**e**) BPS+SMA filter; (**f**) WA filter.

By analyzing the raw PPG signal according to the distances, the amplitude becomes smaller and the noise increases as the distance between the user and sensor increases. If the noise is significant, the amplitude of the near-distance data becomes similar to the amplitude of the far-distance data. Therefore, it is important to minimize this effect when estimating the distance between the user and sensor. Figure 8b, Figure 9b and Figure 10b presents results obtained by the Kalman filter. Because the Kalman filter is a recursive filter based on the original data, no significant differences exist between the raw PPG signal and filtered data. However, the values of raw- and Kalman-filtered data differ from each other, and large noises are discarded certainly. STFT results demonstrate that Figure 10c, the spectrogram for 0.8 mm, differs from Figure 8c, a spectrogram for 0 mm; however, Figure 8c and Figure 9c do not differ significantly. The modified average filter exerts more influence when the average of amplitudes is low because the filter is based on the average. The noise disappears when comparing Figure 10a,d; however, it is not effective for large amplitudes, as illustrated in Figure 8a. Figure 8e, Figure 9e and Figure 10e present the results of the BPS+SMA filter. Because SMA filter was applied after noise reduction, the overall amplitude was significantly decreased. In addition, the relationship between data before and after disappeared. Figure 8f, Figure 9f and Figure 10f show the results of the proposed filter. Although the waveform appears to converge to one value, it is filtered while maintaining the relationship according to all distance criteria.

To verify the suitability of MobileNet for the distance estimation, we employed Intellino, LeNet-5, and a calculation method using the difference between amplitudes without AI. Intellino is an AI with a distance-calculation-based k-neighbor nearest algorithm, not a layer architecture [32]. By reducing the multiplier with the Manhattan distance, the suitability for the embedded system was verified. The accuracy of the audio signal and image data of Intellino was measured at 0.91 and 0.94, respectively [31,33]. Intellino is possible to experiment by freely reconfiguring the size of the input data and the number of neuron cells using the simulator [34]. LeNet-5 is a representative AI for optical character recognition, which has a seven-layer CNN architecture [28]. The convolution layers, sub-sampling layers, and the fully-connected layer are included. Because the size of output data decreases as the sub-sampling layers exist, the minimum size of input data is 32 × 32. Accordingly, we only utilized 120 and 60 as signal lengths. We analyzed the accuracy of the proposed algorithm via the combinations of various filters and four distance estimation methods; the obtained results are presented in Table 1, Table 2, Table 3 and Table 4. Intel-core i5-2500 CPU and 6GB RAM were configured for performance analysis. For the four distance-estimation methods, the tables demonstrate that the accuracy of the WA filter is higher than the accuracy of other filters. Among all the combinations of the filters and distance estimation methods, Intellino exhibits the highest accuracy. However, as presented in Table 5, the inference time is large compared to MobileNet.

We analyzed other metrics such as precision, recall, and f1 score for MobileNet. Precision is the ratio of what is actually true to what the model classifies as true. Recall is the ratio of what is actually true to what the model predicts as true. The F1 score is the harmonic mean of precision and recall. The Precision, recall, and f1 score of MobileNet for the WA filtering data are shown in Table 6. Similar to the results of accuracy, precision, recall, and f1 score were the highest when the signal length was 15. In conclusion, the combination of WA filter and MobileNet for distance estimation achieves high accuracy and practical inference time.

In addition, we verified the proposed algorithm for PPG signals measured at wrists. Dataset obtained from wrists comprised 54,000 sampling points. MobileNet was trained with finger and wrist datasets and was inferenced with wrist dataset. The amplitudes according to the distance of the PPG signal extracted from the wrists were not clearly different from the PPG signal extracted from fingers. As a result, the accuracy, precision, recall, and f1 score were 80.7%, 80.7%, 81.0%, and 80.8, respectively, when the signal length was 20.

## 5. Conclusions

In this paper, we proposed a WA-filter-based distance-estimation algorithm between the user and sensor for PPG signals of TWS. To implement the proposed algorithm, we designed a PPG monitoring testbed, WA filter, and MobileNet. Among them, the WA filter was applied to reduce the noise of raw data as the PPG signals are biological data with several variables. To verify the proposed algorithm, we employed the Kalman filter, STFT, the modified average filter, the BPS+SMA filter, and other AI such as Intellino and LeNet-5. We set three criteria for distance estimation and analyzed the accuracy and inference time according to the combination of various filters and AI. The combination of the WA filter and Intellino exhibited the highest accuracy of 94.6% when the signal length was 10; however, the inference time was 126.462 ms. In contrast, The proposed algorithm showed the highest accuracy of 92.5% and the inference time was 1.561 ms. Furthermore, we assessed the proposed algorithm for the PPG data obtained at wrists. The highest accuracy was 80.7% when the signal length was 20.

## Figures and Tables

**Table 1:** Accuracy of Intellino for filtered PPG data.

Filter	Signal Length					
	120	60	30	20	15	10
Raw data	80.7%	87.6%	90.7%	90.4%	91.0%	90.3%
Kalman	80.1%	88.0%	90.4%	90.2%	90.9%	90.0%
STFT	37.6%	39.8%	40.8%	39.4%	38.8%	37.3%
Modified average	81.6%	89.0%	91.6%	92.0%	92.3%	92.2%
BPS + SMA	62.5%	71.3%	73.5%	73.6%	74.0%	71.1%
WA	81.1%	87.0%	90.7%	93.4%	93.9%	94.6%

**Table 2:** Accuracy of computing using the difference between amplitudes for filtered PPG data.

Filter	Signal Length					
	120	60	30	20	15	10
Raw data	83.6%	82.7%	83.1%	83.4%	83.2%	82.8%
Kalman	83.7%	82.6%	82.9%	83.2%	82.7%	82.7%
STFT	23.9%	25.5%	27.0%	28.2%	29.1%	30.0%
Modified average	86.3%	85.4%	84.9%	84.7%	84.5%	84.4%
BPS + SMA	83.7%	82.4%	80.4%	78.1%	75.9%	74.8%
WA	76.4%	76.0%	75.7%	76.5%	73.9%	75.9%

**Table 3:** Accuracy of LeNet-5 for filtered PPG data.

Filter	Signal Length	
	120	60
Raw data	67.8%	75.6%
Kalman	69.3%	76.5%
STFT	39.5%	41.0%
Modified average	70.4%	78.8%
BPS + SMA	47.2%	50.5%
WA	78.4%	71.5%

**Table 4:** Accuracy of MobileNet for filtered PPG data.

Filter	Signal Length					
	120	60	30	20	15	10
Raw data	88.4%	86.9%	89.4%	90.1%	87.3%	88.1%
Kalman	90.3%	83.6%	89.6%	86.5%	89.3%	87.4%
STFT	40.7%	37.7%	38.8%	37.6%	36.3%	36.2%
Modified average	90.9%	90.4%	90.8%	89.0%	89.7%	89.4%
BPS + SMA	74.8%	77.0%	77.4%	74.7%	73.3%	74.1%
WA	90.3%	90.8%	90.5%	91.2%	92.5%	92.2%

**Table 5:** Inference time of Intellino and MobileNet with WA filter [ms].

Filter	Signal Length					
	120	60	30	20	15	10
Intellino	0.987	3.612	14.037	31.918	54.806	126.462
MobileNet	0.310	0.362	0.535	0.918	1.397	1.561

**Table 6:** Other metrics of MobileNet for WA filtering result.

Metrics	Signal Length					
	120	60	30	20	15	10
Precision	89.7%	90.3%	90.9%	92.0%	92.6%	92.0%
Recall	90.0%	90.5%	91.3%	92.1%	92.8%	92.5%
F1 Score	0.899	0.904	0.911	0.921	0.927	0.922

## Data Availability

Not applicable.
